# Determinants and preventive practices of midwives and physicians toward vertical transmission of hepatitis B in Ghana: a cross-sectional survey

**DOI:** 10.11604/pamj.2022.43.183.31794

**Published:** 2022-12-08

**Authors:** Charles Ampong Adjei, Gilbert Ti-enkawol Nachinab, Fidelis Atibila, Michael Ansah-Nyarko, Josephine Mpomaa Kyei, Peter Kwabena Fosu

**Affiliations:** 1 Department of Community Health Nursing, School of Nursing and Midwifery, University of Ghana, Accra, Ghana,; 2 Department of Midwifery, University for Development Studies of Ghana, Tamale, Ghana,; 3 Department of Nursing, Valley View University, Techiman, Ghana,; 4 Department of Research, Ghana Tertiary Education Commission, Accra, Ghana,; 5 Department of Medicine, The Trust Hospital, Accra, Ghana

**Keywords:** Hepatitis B, prevention of mother-to-child transmission, midwives, physicians, Ghana

## Abstract

**Introduction:**

countries in sub-Saharan Africa, including Ghana, are disproportionately affected by hepatitis B viral (HBV) infection. In these areas, mother-to-child transmission (MTCT) is an essential mode of HBV transmission. Evidently, timely hepatitis B birth dose vaccination remains an effective preventive intervention against MTCT of HBV. Considering that midwives and physicians are the primary care providers of newborns in Ghana, we sought to examine their preventive practices toward vertical transmission of HBV in the eastern region of Ghana.

**Methods:**

a cross-sectional survey was conducted with 126 healthcare providers (HCP; midwives and physicians). The participants were conveniently recruited from one regional hospital and four district hospitals. Statistical significance was set at 0.05 alpha level.

**Results:**

the findings indicate that 42.9% (n = 54) of HCPs' prevention of mother to child transmission (PMTCT) practices for hepatitis B were good (X^2^ = 2.57, p > 0.05). Explicitly, 79% indicated screening all pregnant women for hepatitis B as part of antenatal care (X^2^ = 41.14, p < 0.001). Additionally, about half of the participants (52.4%) reported providing pre-test counselling (X^2^ = 0.29, p > 0.05), whereas one-third (33%) reported routinely administering a birth dose of the hepatitis B vaccine to neonates of mothers with hepatitis B (X^2^ = 14.00, p < 0.001). However, only 37% reported administering the hepatitis B vaccine to newborns within 12 hours of birth (X^2^ = 9.18, p < 0.01). The binary logistic regression analyses identified training as the only significant predictor of good practice on PMTCT of hepatitis B at the 5% level (Wald = 3.91, p =0.05).

**Conclusion:**

given that more than half of the participants in the study area had incorrect PMTCT practices for hepatitis B, it is imperative that a series of workshops on hepatitis B be done for healthcare providers in Ghana. In addition, hepatitis B birth dose vaccine must be incorporated into the ´Expanded Programme on Immunisation´ to remove the cost that acts as a barrier to access.

## Introduction

Hepatitis B viral (HBV) infection is a major health challenge with a prevalence estimate of 298 million worldwide [1]. The high burden of HBV in Africa [1,2], has raised concerns about the need to consider hepatitis B as a neglected tropical disease [3]. Evidence suggests that perinatal route is an important mode of HBV transmission, particularly in high endemic countries in Africa [1,4]. Given that HBV is highly infectious [1,4,5], early detection of the virus through screening, especially during pregnancy is very crucial [6]. About 80-90% of infants exposed to HBV in their first year of life may develop chronic hepatitis B and further risk the chance of developing liver cirrhosis, liver cancer, and liver failure later in life [1]. Fortunately, an effective vaccine, including the hepatitis B vaccine and hepatitis B immunoglobulin against HBV exist. This vaccine has shown to prevent MTCT of HBV when administered to newborns of HBV positive mothers within 12 hours after birth [6]. About 75-90% of HBV infections can be prevented following neonatal immunisation with either hepatitis B vaccine alone or in combination with hepatitis B immunoglobulin [7].

Although Ghana has HBV prevalence (i.e. HBsAg positive) of 12.3% [8], administration of hepatitis B vaccine birth-dose is not a national policy [9]. Currently, hepatitis B vaccination (pentavalent vaccine) is routinely provided for newborns at 6, 10, and 14 weeks after birth [9,10]. This immunisation schedule raises the concern of vulnerability of newborns to HBV positive mothers within the first six weeks of delivery. Per the health system structure in Ghana, midwives and physicians are the primary healthcare providers for pregnant women and their newborns. Specifically, newborns spend the first 12h of their lives in the health facilities after birth and therefore, could benefit from PMTCT interventions if initiated by the HCPs [9] following childbirth. We contended that these cadre of staff could ensure timely administration of hepatitis B birth-dose vaccination per the Centers for Disease Control and Prevention (CDC) guideline [6]. We therefore, sought to examine the practices and the factors that influence the practices of midwives and physicians toward PMTCT in the eastern region of Ghana.

## Methods

**Study design:** a quantitative approach [11] using a cross-sectional survey was used [12].

**Study setting:** this study was conducted in the eastern part of Ghana. According to the most recent population and housing census report, about 2,633,154 people reside in the eastern region of Ghana [13]. The region has a regional hospital and a number of districts, and sub-district health facilities owned by the Government of Ghana and the Christian Health Association of Ghana (CHAG). This study was conducted in the only regional hospital and four district hospitals in the region. This study is part of a larger study, but the findings as reported here are about the preventive practices of midwives and physicians [9].

**Study participants:** participants were included if they were full-time employees of the selected health facilities with at least six (6) months working experience in midwifery or medicine. Additionally, participants were considered eligible if they were providing either maternal or neonatal care in any of the selected health facilities and consented to participate. Midwives and physicians were excluded if they were doing national service or internship respectively.

**Sampling technique:** convenience sampling technique was used to recruit participants who met the inclusion criteria [13]. The physicians were approached in their consulting rooms following a prior notice a week to data collection. Also, the midwives were contacted in their various place of work including the labour ward, antenatal unit, lying-in ward, and post-natal unit. Data collection spanned between March and September, 2017.

**Outcome measures:** the primary outcome of the study was the preventive practices of the midwives and physicians toward vertical transmission of hepatitis B.

**Data collection:** we used a semi-structured self-administered questionnaire to collect participants' response to the research questions. The questionnaire was developed by experts in the field, including gastroenterologist, obstetric and gynaecologist specialist, and a hepatitis researcher. The questionnaire was pre-tested using five physicians and ten midwives. Items reported to be unclear to the participants were revised accordingly. Cronbach´s alpha reliability coefficient was established after the revised questionnaire was administered to 30 participants and found to be 0.74 which was considered acceptable [14]. Six (6) questions were asked on levels of practice on PMTCT of HBV. Practice was categorised into good and bad. This was based on the standard measure of the Ghana Education Service 50% cut off rating for pass or fail in an examination.

**Sample size determination:** Yamane [15] formula for calculating sample size at a precision level of 0.05 was used to estimate the sample size for this study. A total of 120 participants was the minimum sample size required. However, 126 questionnaires were retrieved, out of 130 questionnaires administered, representing a response rate of 97%.

**Statistical analysis:** we analysed the data using the IBM Statistical Product and Service Solutions (IBM-SPSS) version 24.0. Descriptive statistics such as means, standard deviation, and percentages were examined. Inferential statistics, including Chi-square, and binary logistic regression, were performed. A p-value of 0.05 alpha level was considered statistically significant.

**Informed consent:** written informed consent was obtained from every participants after explaining the purpose, confidentiality information, and the right to withdraw from the study.

**Ethical considerations:** ethical approval was obtained from the Ghana Health Service ethical review committee (approval number GHS-ERC 13/07/15). We also obtained permission from the management of the selected facilities.

## Results

**Sociodemographic characteristics**: we recruited 126 healthcare providers. Physicians were thirty-nine (n=39, 31%) and midwives were eighty-seven (69.0%). A higher proportion of the participants were females (n=100, 79.4%) and the remaining participants were males (n=26, 20.6%). With respect to the type of health facilities, 30.2% (n=38) of the participants were from the regional government hospital, 42 (33.3%) were from the district government hospitals. Out of the total number of participants from the district government hospitals, 24 (19%) were from Oda Government Hospital and 18 (14.3%) were from the Nsawam Government Hospital. Overall, 46 (36.5%) were from the district hospital. Specifically, 26 (20.6%) of the participants were from St. Joseph Hospital and 20 (15.9%) were from St. Martins Hospitals. Table 1 presents the summary of the descriptive statistics in details and published elsewhere [9].

**Table 1:** summary of sociodemographic characteristics of participants

Variables	Frequency	Percentage (%)
**Profession**		
Midwives	87	69.0
Physician	39	31.0
**Sex**		
Male	26	20.6
Female	100	79.4
**Age**		
20-25 years	22	17.5
26-30 year	56	44.4
31-35 years	22	17.5
36-40 years	12	9.5
41-45 years	1	0.8
46-50 years	3	2.4
51 years and above	10	7.9
**Qualification**		
Certificate	19	15.1
Diploma	68	54.0
First degree	25	19.8
Post graduate	14	11.1
**Years of service**		
Less than 1 year	25	19.8
1-2 years	54	42.8
3-4 years	27	21.4
5-6 years	6	4.8
7-8 years	6	4.8
9-10 years	1	0.8
11 years and above	7	5.6
**Unit of work**		
Antenatal care	26	20.6
Labor ward	54	42.9
Lying in ward	14	11.1
Post-natal unit	7	5.6
All units	25	19.8
**Type of health facility**		
Regional hospital	38	30.2
District government hospital	42	33.3
Oda hospital	24	19.0
Nsawam hospital	18	14.3
District mission hospital	46	36.5
St. Joseph Hospital	26	20.6
St. Martin Hospital	20	15.9

** p<0.01; ***p<0.001

In Table 2, physicians and midwives were examined on practices of PMTCT of HBV infection within their facility. Out of the 126 professionals, ninety-nine (99) representing 78.6% indicated that they screen every pregnant woman for hepatitis B infection during the antenatal period all the time and 27 representing 21.4% said they rarely do that. The difference is statistically significant (X2 = 41.14, p < 0.001). In addition, 66 representing 52.4% said they all the time offer pre-counselling on hepatitis B to pregnant women before screening whilst 60 representing 47.6% rarely do it (X2 = 0.29, p > 0.05). Also, only 42 representing 33.3% all the time vaccinate newborns of HBV carrier mothers with only hepatitis B vaccine and 84 representing 66.7% do otherwise (X2 = 14.00, p < 0.001). More so, 45 representing 35.7% vaccinate with hepatitis B vaccine alongside hepatitis B immunoglobulin to newborns of hepatitis B positive mothers whiles 81 representing 64.3% do not (X2 = 10.29, p < 0.001). Furthermore, only 46 representing 36.5% administer hepatitis B vaccine and hepatitis B immunoglobulin to newborns of HBV carriers within 12 hours after birth whilst 80 representing 63.5 do not (X2 = 9.18, p < 0.01). Finally, only 6 representing 4.8% of midwives and physicians when encounter a woman with unknown status do not assume the person is negative and therefore test to identify the status whiles a greater number of 120 representing 95.2% assumes that such a person is negative hence no test is needed (X2 = 103.14, p < 0.001).

**Table 2:** summary of mean, standard deviation, descriptive and Chi-square analysis of practice on PMTCT of HBV infection

Practice items	Mean (SD)	Correct (%)	Incorrect (%)	X^2^
I screen every pregnant woman for hepatitis B infection during the antenatal period	0.79 (±0.412)	99 (78.6)	27 (21.4)	41.14***
I offer pre-counselling on hepatitis B to pregnant women before screening them	0.52 (±0.501)	66 (52.4)	60 (47.6)	0.29
I vaccinate newborns of HBV carrier mothers with only hepatitis B vaccine without immunoglobulin	0.33 (±0.473)	84 (66.7)	42 (33.3)	14.00***
I vaccinate with hepatitis B vaccine alongside hepatitis B immunoglobulin to newborns of hepatitis B carrier mothers	0.36 (±0.481)	45 (35.7)	81 (64.3)	10.29***
I administer hepatitis B vaccine and hepatitis B immunoglobulin to newborns of HBV carriers within 12 hours after birth	0.37 (±0.483)	46 (36.5)	80 (63.5)	9.18**
When I encounter a woman with unknown status, I assumed the person is negative and therefore no test is needed	0.05 (±0.214)	6 (4.8)	120 (95.2)	103.14***

** p<0.01; ***p<0.001

In Table 3, 54 respondents representing 42.9% engaged in good practices on PMTCT of HBV infection within their facility and 72 representing 57.1% of them engaged in bad practices (Chi-square = 2.57, p > 0.05). Meaning more people were found to engage in bad practices at the hospital whilst just a hand-full engage in good practices. Even though there is no statistically significant difference, the greater number of physicians and midwives doing the wrong thing within the health facility is quite worrying and poses a threat to health delivery within the region.

**Table 3:** summary of descriptive statistics and Chi-square analysis of levels of practices on PMTCT of HBV infection

Levels	Frequency (%)	Chi-square
Good	54 (42.9)	2.57
Bad	72 (57.1)	

N = 126; note: correct practices were coded 1 and incorrect practices were coded 0; on the practices scale, there was a total of 6 items, so the minimum score expected is 0 and the maximum score is 6; the researchers therefore used the standard measure of rating someone to have passed or fail an exam by the Ghana Education Service of 50% cut off to categorize practice into good and bad; therefore a score of 3 to 6 was considered good practice and score below 3 were considered to be bad practice

The binary logistic indicates that training is a significant predictor of practice on PMTCT of HBV infection (Chi-square = 6.97, df = 5 and p = 0.22). The other four predictors profession, stocks of vaccine, availability of immunoglobulin and protocol are not significant. All the five predictors explained 7.2% of the variability in practice. Training is significant at 5% level (training Wald = 3.91, p =0.05). The odds ratio (OR) for training 0.34 (95% C.I., 0.12-0.99). The model correctly predicted 66.7% cases where practice on PMTCT of HBV infection was bad and 51.9% cases where practice on PMTCT of HBV infection was good, giving an overall prediction rate of 60.3%.

From the analysis in Table 4, it was observed that inadequate training on HBV infection is a major predictor of the bad practices on PMTCT of HBV infection among both physicians and midwives in the three facilities in the eastern region.

**Table 4:** summary of binary logistic regression of profession, factors and practice on PMTCT of HBV infection

		B	S.E.	Wald	df	Sig.	Exp(B)	95% C.I. for Exp(B)	
								Lower	Upper
Step 1^a^	Profession	0.08	0.42	0.04	1	0.85	1.08	0.48	2.46
	Training	-1.07	0.54	3.91	1	0.05	0.34	0.12	0.99
	Vaccine stock	-0.53	0.61	0.76	1	0.38	0.59	0.18	1.95
	Immunoglobulin	1.13	0.63	3.22	1	0.07	3.10	0.90	10.67
	Protocol	-0.00	0.43	0.00	1	0.99	1.00	0.43	2.33
	Constant	-0.52	0.43	1.46	1	0.23	0.59		

a variable(s) entered on step 1: profession, training, vaccine stock, immunoglobulin, protocol

## Discussion

This study examined the practices of physicians and midwives in the eastern region of Ghana. Consistent with other studies [16,17], our findings showed a prevalence of incorrect practices on PMTCT of HBV among the physicians and midwives. Explicitly, about 78.6% (n=99) of our participants indicated that they routinely screen pregnant women for HBV during the ante-natal period. Although this observation is appreciable, it also suggests a missed opportunity by the HCPs to diagnose and link potential HBV positive mothers to care and their newborns to PMTCT interventions. Comparatively, the HBV screening practices of our participants were below that of midwives and obstetricians in United State of America where about 97% of the providers were found to have tested pregnant women for HBV [18]. Leaning on literature in the study area [9], it can be contended that screening of pregnant women during antenatal care (ANC) was not a protocol in some of the selected health facilities. This could present a negative implication for PMTCT given that a substantial number of babies born to these mothers might not be vaccinated against HBV at birth in line with the recommendation by CDC and World Health Organization (WHO) [1,6].

In addition to the poor HBV screening practices recorded, pre-counselling on HBV for pregnant women was done by only 52.4% of our participants. The most likely explanation is that, perhaps, the HCPs were not confident enough to offer accurate information on HBV to their patients [19]. This assertion could also be supported by a previous study that reported knowledge gap on MTCT of HBV among HCPs in the study area [9]. Our finding is in line with a study that evaluated current levels of hepatitis B-related knowledge and clinical practice among perinatal nurses in California and found 51% of the participants providing hepatitis B related information to their patients [20].

Furthermore, we identified that only 35.7% (n=45) of the HCPs vaccinated newborns of HBV positive mothers with hepatitis B vaccine in combination with hepatitis B immunoglobulin. This is an unacceptable response to HBV in an endemic country like Ghana where about 12.3% of the population are infected [8], and 10.2% of pregnant women were found to be HBV positive in a recent study [21]. It can be argued that the cost of hepatitis B vaccine and hepatitis B immunoglobulin is too high for the average Ghanaian to afford since these services are not part of the Expanded Programme on Immunisation (EPI) and the costs are not included in the National Health Insurance Scheme (NHIS). This is therefore disconcerting, and it could threaten Ghana´s chances of meeting the global target of eliminating HBV by 2030 [22]. However, this is not peculiar to Ghana as Goncalves and Goncalves [17] also reported that a number of HCPs failed to request for hepatitis B vaccine for newborns in Amazonas State. In addition, the high cost of the hepatitis B vaccine for pregnant women that act as a barrier to vaccination of newborns at birth has also been observed elsewhere [23].

In addition to the gaps in practices mentioned above, the binary logistic regression findings revealed training as a predictor of good practices for PMTCT of HBV. This therefore, underscores the relevance of capacity training for HCPs by the institutions responsible including the Nursing and Midwifery Council (NMC) of Ghana and the Medical and Dental Council (MDC). The training can be in a form a workshop and provision of hepatitis B resources to the HCPs. This is critical in the study area since, in a previous study, 49.2% of physicians and midwives in the region indicated that they had never attended a workshop on HBV after their formal training [9].

This study has some theoretical implications. In terms of theory, this study adds to the limited studies on the practices of HCPs on PMTCT of HBV in Africa. In terms of practice, given the incorrect prevalence of hepatitis B practices among the HCPs, we feel that capacity training of HCPs in the area is needed. Also, based on the findings of inadequate vaccination of newborns to HBV mothers by the HCPs, we recommend the integration of hepatitis B birth dose vaccination into the Expanded Programme on Immunisation to remove the cost that perhaps act as a barrier to its provision. We also believe that pre-counselling and post counselling on HBV information for patients should be part of HCPs core functions in Ghana health delivery system to manage some of the psychological challenges faced by individuals following their HBV diagnosis [24].

**Strength and limitations:** although this study is one of the first studies to determine the practices of physicians and midwives on PMTCT in Ghana, it should be viewed in light of some few limitations. First, we could not establish the reliability of the participants´ response because of the self-report approach used. Second, considering the fact that we used only few health facilities, one has to be cautious in generalising the study findings to the entire regions of Ghana. Also, the crosss-sectional-survey used could not establish cause and effect relationship.

## Conclusion

This study sought to identify the determinants and preventive practices of physicians and midwives toward vertical transmission of HBV in the eastern region of Ghana. The outcome of the study highlights the need for Ghana to strengthen its response to the burden of HBV in order to meet the global target of eliminating HBV as a public health threat by 2030.

### What is known about this topic

- Hepatitis B remains a public health threat in Africa with continual occurrence of vertical transmission;
- Timely administration of hepatitis B vaccine and immunoglobulin reduce the vulnerability of newborns of mothers with hepatitis B infection;
- Healthcare providers, particularly midwives and physicians play essential roles in the prevention of hepatitis B transmission from infected mothers to their newborns within the first 24hr following birth.

### What this study adds

- A significant number of midwives and physicians do not have good preventive practices toward mother-to-child transmission of hepatitis B;
- Capacity training of midwives and physicians on vertical transmission of hepatitis B has been shown as important predictor of preventive practices.
